# Comparison of Neoadjuvant Chemotherapy With FLOT and Modified DCF Regimens in Nonmetastatic Gastric Adenocarcinoma

**DOI:** 10.1002/cnr2.70247

**Published:** 2025-06-02

**Authors:** Mehdi Pourghasemian, Maryam Salimi, Effat Iranijam, Mohammad Negaresh

**Affiliations:** ^1^ Department of Internal Medicine, Hematology and Oncology Division School of Medicine, Ardabil University of Medical Sciences Ardabil Iran; ^2^ Department of Internal Medicine School of Medicine, Ardabil University of Medical Sciences Ardabil Iran

**Keywords:** FLOT, modified DCF, neoadjuvant chemotherapy, nonmetastatic gastric adenocarcinoma

## Abstract

**Backgrounds:**

Gastric adenocarcinoma is a common and severe type of malignancy. Treatment for advanced cases involves neoadjuvant chemotherapy before surgery and adjuvant chemotherapy if needed.

**Aims:**

In this study, a comparison of two regimens of FLOT and mDCF has been conducted with regard to pathological and radiological response, as well as complications.

**Methods and Results:**

The medical records of patients diagnosed with nonmetastatic gastric adenocarcinoma who randomly received therapy with either FLOT or mDCF regimens were studied. The two groups were compared regarding complications, radiological response based on RECIST Ver 1.1 criteria, the chance of successful gastrectomy, pathological response based on the TRG scale, and downstaging of the gastric adenocarcinoma following chemotherapy. In total, 90 patients were studied. 40 patients were treated with the mDCF regimen, while 50 received the FLOT regimen. The mDCF group experienced more side effects, and the FLOT group had higher response rates and a greater percentage of patients who underwent surgery with clear margins. Additionally, patients receiving FLOT treatment showed greater lymphatic involvement, tissue invasion, and disease stage improvement.

**Conclusion:**

According to the study, patients with limited local invasion gastric adenocarcinoma who are eligible for surgery may benefit more from the FLOT neoadjuvant regimen than the mDCF regimen. The FLOT regimen proves to be more efficient and has fewer complications.

## 1 Introduction

Gastric cancer (GC) is the fifth most common cancer in the world. Additionally, this disease is the second leading cause of cancer‐related deaths globally [1, 2]. Developing countries, Eastern Europe, South America, and Asia account for two‐thirds of the prevalence of GC [3]. The probability of this malignancy is higher in urban societies and groups with lower economic status. GC rarely occurs before the age of 40, and after this age, the incidence increases linearly, so the maximum incidence is in the seventh decade of life. Men are affected twice as often as women [4]. GC is the second most common cancer in Iran. Studies show that it has a high prevalence in the west and northwest regions, particularly in East Azerbaijan and Ardabil [5, 6]. Lauren classified GC into four subtypes: intestinal, diffuse, mixed, and unclassified. In another classification, it is classified as signet ring‐cell carcinoma, adenocarcinoma, and undifferentiated carcinoma. Adenocarcinoma accounts for 90% of cases [7]. From 1996 to 2002, the average 5‐year survival of GC patients in America was only 24% [8]. The standard treatment for operable GC is completely removing the macroscopic and microscopic parts of the disease through surgery. However, the probability of local‐regional recurrence after surgery is around 80%. In the United States, most cases are diagnosed late, when they reach the advanced stages (stages 3–4), in which case the survival rate is less than 20%. In these stages, although palliative chemotherapy is used to control the symptoms, the survival rate does not improve significantly [9].

Different staging methods are suggested for GC, one of which is TNM staging by the American Joint Committee on Cancer (AJCC). According to this staging method, stage 0 (TisN0M0) is defined as no nodal involvement, and the disease is limited to the mucosa. Stage IA (T1N0M0) is defined as having no nodal involvement with invasion into the lamina propria or submucosa. Stage IB (T2N0M0 or T1N1M0) is characterized by no nodal involvement and invasion of the muscularis propria. Stage II (T1N2M0, T2N1M0, T3N0M0, or T2N2M0) is defined as nodal involvement with invasion beyond the mucosa but within the wall, or no nodal involvement with extension through the wall. Stage IIIA (T3N1‐2M0) indicates nodal involvement with invasion of the muscularis propria or through the wall, while IIIB (T4N0‐1M0) signifies no nodal involvement with adherence to surrounding tissue. Stage IIIC (T4N2‐3M0 or T3N3M0) involves more than three nodes and either invasion of the serosa or adjacent structures, or seven or more positive nodes with penetration of the wall without invading the serosa or adjacent structures. Stage IV (T4N2M0 or T1‐4N0‐2M1) indicates nodal positivity, adherence to surrounding tissue, or distant metastases [10].

Neoadjuvant chemotherapy was first used to treat locally advanced and inoperable GC in 1989 [9]. The MAGIC study in England and the ACCORD study in France evaluated the effectiveness of two chemotherapy regimens, ECF (on the first day, Epirubicin 50 and Cisplatin 60 mg/m^2^, along with continuous infusion of Fluorouracil‐5, 200 mg/m^2^/day from day 1 to day 21) and CF (On the first day Cisplatin 100 mg/m^2^, and an infusion of 1000 mg/m^2^ Fluorouracil‐5 from day 1 to 5), both before and after surgery. The results of these studies were successful and led to the acceptance of this method as an effective chemotherapy treatment in Europe [11, 12, 13, 14]. Currently, in stages higher than T2, neoadjuvant chemotherapy before and adjuvant chemotherapy after surgery are recommended.

Studies show a better response for GC cases treated with three‐drug regimens and combination therapy [15, 16, 17]. The most neoadjuvant chemotherapy regimens used in recent years for GC patients have been ECF or DCF (5 days of docetaxel 75 mg/m^2^ day, Cisplatin 75 mg/m^2^, and Fluorouracil 750 mg/m^2^/day) regimen for three cycles. Compared to the CF regimen, a greater rate of improvement in time to progression, quality of life, and overall survival (OS) has been observed with the neoadjuvant DCF regimen [18]. Recently, the FLOT regimen consisting of 50 mg/m^2^ of docetaxel on day 1, 85 mg/m^2^ of oxaliplatin on day 1, 200 mg/m^2^ of leucovorin on day 1, and 2600 mg/m^2^ of fluorouracil continuously infused over 24 h on day 1 has also been proposed as a new and effective chemotherapy regimen in patients who can tolerate the 3‐drug regimen. Studies indicate that compared to surgical treatment alone, it shows a higher OS and three‐year survival rate [19].

Considering the high rate of GC patients in the Ardabil region, the acceptable statistical population, and the limited studies conducted about the comparison of FLOT and DCF regimens, this study compares these two treatment regimens in terms of pathological response, radiological response, and adverse effects.

## 2 Materials and Methods

This study is designed as a retrospective study in which patients who were referred to Imam Khomeini Hospital in Ardabil from April 2018 to March 2019 with the diagnosis of locally advanced GC and underwent neoadjuvant chemotherapy, followed by radical gastrectomy, were investigated. For this study, the medical records of patients were analyzed. Inclusion criteria for our study included a biopsy‐confirmed gastric adenocarcinoma, deep mucosal layer involvement of T_IB_‐T_IV_ based on EUS Staging, age less than 75 years, Eastern Cooperative Oncology Group (ECOG) Score 0 to 2, more than 4000 cu/mm of white blood cells, more than 100 × 10^9^ cells per L of platelet count, total bilirubin of less than 1.2 mg/dL, alanine aminotransferase (ALT) or aspartate aminotransferase (AST) of less than 40 IU/L, left ventricular ejection fraction of more than 50%, and serum creatinine level of less than 1.4 mg/dL, absence of metastasis or, if present, metastasis limited to retroperitoneal lymph nodes. Patients with a previous history of other cancers, a history of receiving other treatment modalities such as radiotherapy, the presence of severe co‐morbidities such as heart, kidney, and liver failure, intolerance to a three‐drug chemotherapy regimen, abnormal hematological indexes except for anemia, very severe toxicity caused by treatment, patient noncooperation, and death during chemotherapy were excluded from the study. Consent forms were obtained from all patients before starting the treatment regimen and in all stages of treatment after a complete verbal explanation of each stage.

All the patients were subjected to initial examination for cancer staging, including an enhanced chest and abdominal CT scan, endoscopy, EUS, complete blood cell differentiation and count, and complete kidney and liver function tests. After the initial examinations, the patients were studied in two groups. The first group received treatment with four courses of the FLOT regimen (60 mg/m^2^ of Docetaxel in short infusion on the first day, 200 mg/m^2^ of Leucovorin, 85 mg/m^2^ of Oxaliplatin, and 2600 mg/m^2^ Fluorouracil‐5, during 48‐h infusion every 2 weeks). The second group was treated with three courses of modified DCF (mDCF) regimen (75 mg/m^2^ of Docetaxel, 75 mg/m^2^ of Cisplatin on the first day, 750 mg/m^2^ infusion of Fluorouracil‐5 during 24 h from the first day to the third day every 3 weeks). In this study, we modified the DCF regimen by administering 750 mg/m^2^ of Fluorouracil‐5 over 3 days instead of five. Antiemetic prophylaxis and other supportive treatments were also provided according to treatment protocols.

After completing the abovementioned treatment, the patients underwent a CT scan 4 weeks later. A total gastrectomy was performed if they responded positively to the treatment based on the 1.1 version of RECIST criteria [19] and showed no signs of distant metastasis. The 1.1 version of RECIST criteria provides a guideline for assessing tumor response based on radiologic findings. According to this guideline, if all of the target lesions disappear and the axis of all pathological lymph nodes decreases to less than 10 mm, the complete response (CR) is reached if the total diameter of the target lesions is at least 30% reduced; partial response (PR) is achieved. In addition, based on this criteria, when the total diameter of target lesions increases by at least 20%, plus at least a 5 mm increase in total size, or one or more new lesions appear, the condition is defined as progressive disease (PD), and stable disease (SD) is defined as the absence of adequate shrinkage to include PR and the lack of sufficient size increase to qualify for PD [20]. It should be noted that in this study, patients with PD and SD were considered nonresponders to treatment. The sample prepared during surgery was examined for the changes in the stage of involved lymph nodes, pathological response based on Tumor regression criteria (TRG) [21], and microscopically margin‐negative resection. Based on research on TRG classification methods, the Mandard method was selected due to its superior ability to assess patient prognosis [22]. The Mandard TRG criteria [23] categorize the response to chemotherapy into five groups. TRG1 indicates a complete response with no viable cancer cells present; TRG2 shows the presence of single cells or small groups of cancer cells; TRG3 demonstrates a higher rate of fibrosis growth compared to cancer growth; TRG4 indicates a higher rate of cancer growth than fibrosis growth, whereas TRG5 denotes no response to chemotherapy. Blood and gastrointestinal complications, more severe than grades 3–4, were evaluated to assess regimen complications. After 8 weeks, an enhanced CT scan and EUS were performed again to assess the lymphatic involvement, the depth of involvement, and the stage of the disease. All data obtained from the two groups of neoadjuvant chemotherapy regimens were compared. Demographic information, age, sex, complications, blood test, pathology, CT scan, and EUS results were recorded for each patient. In comparing drug toxicities according to the Common Terminology Criteria for Adverse Events and common toxicity criteria [24] as well as the Common Toxicity Criteria [25], nephrotoxicity is defined as an increase in creatinine levels above baseline (1.4 mg/dL), and hepatotoxicity is defined as Aspartate aminotransferase or Alanine aminotransferase levels above baseline (40 μ/L). Severe neutropenia is defined as an absolute neutrophilic count (ANC) of less than 1000/μL, while severe thrombocytopenia is defined as platelet levels of less than 50 000/μL. After completion, the information was entered into the software. Quantitative data was analyzed using frequency, standard deviation, and mean. The variables in each group were compared using parametric and nonparametric criteria, specifically Wilcoxon and Chi‐Square tests. For comparison of the variables between the two groups, the Mann–Whitney test was used. A *p*‐value of less than 0.05 is regarded as a statistically significant result. This study was approved by the Ethics Committee of Ardabil University of Medical Sciences with the code IR.ARUMS.REC.1399.572 and was conducted according to the Declaration of Helsinki.

## 3 Results

**FIGURE 1:**
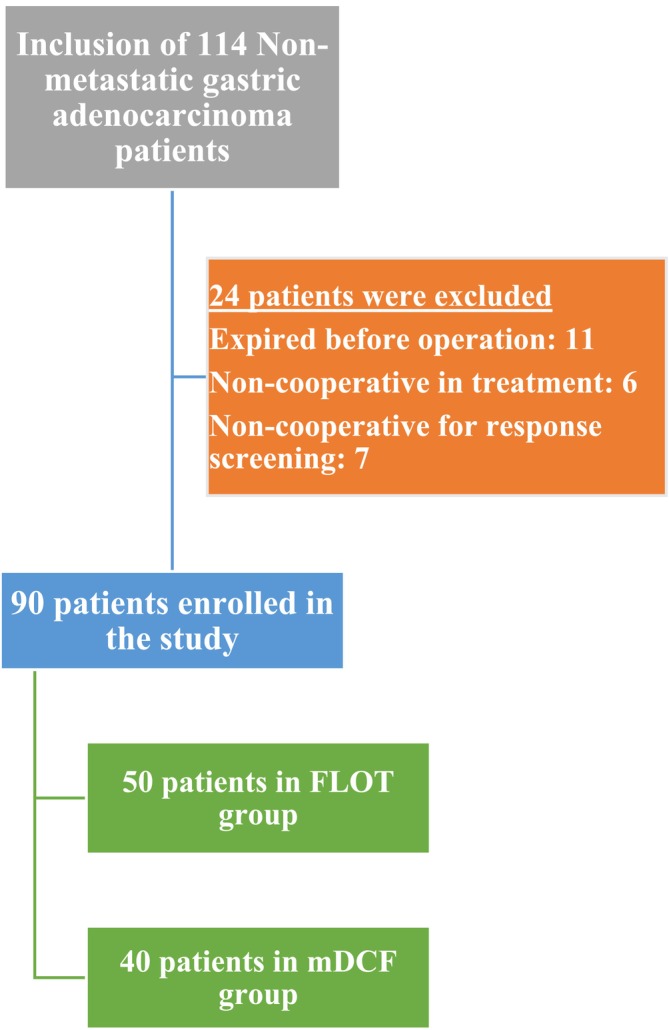
Patient selection and matching flowchart.

At the beginning of the study, 114 patients were involved. However, 24 patients were excluded during the process. Eleven patients passed away before the operation, six could not tolerate the treatment regimen, and seven did not cooperate with response screening. Overall, 90 patients—40 on mDCF and 50 on FLOT—were included in the study. Figure 1 displays the patient selection and matching flowchart.

The patients' average age was 61.09 ± 8.9 years, ranging from a minimum of 35 to a maximum of 75 years. The average age of patients in the mDCF and FLOT groups was 60.37 ± 8.08 and 61.73 ± 9.61 years, respectively. Based on the independent *t*‐test, there were no significant differences in the average age groups (*p* = 0.41). Male patients accounted for 65.55% of cases, with the cardia being the most commonly affected stomach region by GC (61.11%). Additionally, gastric adenocarcinoma of the intestinal type had a higher prevalence (66.66%). Based on the TNM staging [10] during initial examinations, most patients showed deep involvement at T3 and T4 levels, with N2 and N3 lymph node involvement, and stage 2 or 3 (Table 1). The demographic findings of the patients are presented in Table 2.

**TABLE 1:** Comparison of treatment response in two groups of FLOT and mDCF regimens.

Response factors			Neoadjuvant regimen		*p*
			mDCF (*n* = 40)	FLOT (*n* = 50)	
Radiologic response RECIST (Ver 1.1)	Complete response		11 (27.5%)	25 (50%)	0.006
	Partial response		20 (50%)	22 (44%)	
	No response		9 (22.5%)	3 (6%)	
Tumor regression grade (TRG)	Complete or near‐complete response	TRG1	3 (7.5%)	13 (26%)	0.001
		TRG2	3 (7.5%)	15 (30%)	
		TRG3	15 (37.5%)	16 (32%)	
	Relative or no response	TRG4	7 (17.5%)	4 (8%)	
		TRG5	12 (30%)	2 (4%)	
Tumor surgery with a clear margin (R0 resection)	Yes		21 (52.5%)	40 (80%)	0.006
	No		19 (47.5%)	10 (20%)	

Abbreviation: RECIST, Response Evaluation Criteria in Solid Tumors.

**TABLE 2:** Demographic findings of patients in the two groups of the FLOT and mDCF regimen.

Variables		Neoadjuvant regimen		*p*
		mDCF (*n* = 40)	FLOT (*n* = 50)	
Age		60.37 ± 8.08	61.73 ± 9.61	0.41
Gender	Male	25 (62.5%)	34 (68%)	0.58
	Female	15 (37.5%)	16 (32%)	
Involved region	Cardia	25 (62.5%)	30 (60%)	0.81
	Body	15 (37.5%)	20 (40%)	
Type of gastric cancer	Intestinal	25 (62.5%)	35 (70%)	0.45
	Diffuse	15 (37.5%)	15 (30%)	

### 3.1 Toxicity

In a comparison of drug toxicity between the two groups, a significant relationship in nephrotoxicity, neutropenia with or without fever, severe thrombocytopenia, and neuropathy was seen using the Mann–Whitney test (*p* < 0.05). A higher incidence of neutropenia without fever, thrombocytopenia, and kidney complications was found in the mDCF group, while in terms of neuropathy and incidence of febrile neutropenia (taking into account all degrees of neutropenia), the FLOT group presented a significantly higher incidence (*p* < 0.05). Our study found that the most common complications among both groups were nausea, all‐grade neuropathy, and febrile neutropenia. Severe neutropenia (ANC < 1000/micro L) without fever was mostly observed in the mDCF group, with a rate of 62.5% (*p* = 0.003). Febrile neutropenia (ANC < 1500/micro L) was more prevalent in the FLOT group, affecting 76% of participants (*p* = 0.001). The mDCF group exhibited higher rates of severe thrombocytopenia at 52.5% (*p* = 0.004). Additionally, Grade 3 and 4 neuropathy was more common in the FLOT regimen, impacting 14% of patients. No significant differences were seen regarding the gastrointestinal complications in groups (*p* > 0.05) (Table 3).

**TABLE 3:** Comparison of the complications in the two groups of FLOT and mDCF regimens.

Complications		Neoadjuvant regimen		*p*
		mDCF (*n* = 40)	FLOT (*n* = 50)	
Severe neutropenia without fever (ANC < 1000/μL)	Yes	25 (62.5%)	18 (36%)	**0.003**
	No	15 (37.5%)	32 (64%)	
Febrile neutropenia (ANC < 1500/μL)	Yes	17 (42.5%)	38 (76%)	**0.001**
	No	27 (57.5%)	12 (24%)	
Severe thrombocytopenia (Plt < 50 000/μL)	Yes	21 (52.5%)	12 (24%)	**0.004**
	No	19 (47.5%)	38 (76%)	
Neuropathy	No	18 (45%)	4 (8%)	**0.001**
	Grade 1	11 (37.5%)	15 (30%)	
	Grade 2	9 (22.5%)	24 (48%)	
	Grade 3	1 (2.5%)	7 (14%)	
	Grade 4	1 (2.5%)	0	
Nausea	Yes	34 (85%)	37 (74%)	0.206
	No	6 (15%)	13 (36%)	
Diarrhea	Yes	13 (32.5%)	24 (48%)	0.140
	No	37 (67.5%)	26 (52%)	
Hepatotoxicity (AST or ALT > normal upper limit (40 μ/L))	Yes	9 (22.5%)	9 (18%)	0.6
	No	31 (77.5%)	41 (82%)	
Nephrotoxicity (Cr > normal upper limit (1.4 mg/dL))	Yes	16 (40%)	6 (12%)	0.002
	No	24 (60%)	24 (88%)	

*Note:* A *p*‐value of less than 0.05 is considered statistically significant.

Abbreviations: ALT, alanine aminotransferase; ANC, absolute neutrophilic count; AST, aspartate aminotransferase; Cr, creatinine; Plt, platelet.

### 3.2 Radiologic Response

The decrease in lymphatic involvement, tissue invasion, and stage based on radiologic assessment was significantly higher in the FLOT group than in the mDCF group (*p* < 0.05) (Table 4).

**TABLE 4:** Comparison of radiological response in two groups of FLOT and mCDF regimens.

Variables		mDCF (*n* = 40)			FLOT (*n* = 50)		
		Before treatment	After treatment	*p*	Before treatment	After treatment	*p*
Lymph node involvement	N0	4 (10%)	13 (32.5%)	0.035	4 (8%)	25 (50%)	0.001
	N1	18 (45%)	20 (50%)		22 (44%)	24 (48%)	
	N2	14 (35%)	2 (5%)		19 (38%)	1 (2%)	
	N3	4 (10%)	5 (12.5%)		5 (10%)	0 (0)	
Deep mucosal layer involvement	T1	1 (2.5%)	14 (35%)	0.028	0 (0%)	22 (44%)	0.001
	T2	14 (35%)	5 (12.5%)		10 (20%)	18 (36%)	
	T3	15 (37.5%)	12 (30%)		28 (56%)	9 (18%)	
	T4	10 (25%)	9 (22.5%)		12 (24%)	1 (2%)	
Stage	1	0 (0%)	14 (35%)	0.025	0 (0%)	30 (60%)	0.001
	2	23 (57.5%)	14 (35%)		27 (54%)	9 (18%)	
	3	17 (42.5%)	7 (17.5%)		23 (46%)	8 (16%)	
	4	0 (0%)	5 (12.5%)		0 (0%)	3 (6%)	

### 3.3 Treatment Response

The FLOT group showed a significantly higher rate of treatment response based on RECIST and TRG criteria than the mDCF group, according to the Mann–Whitney test (*p* < 0.05). 40 patients (80%) in the FLOT group underwent surgery with clear margins, compared to 21 patients (52.5%) in the mDCF group (*p* = 0.006). According to TRG, partial and complete response (TRG 1, 2, 3) was seen in 44 patients (88%) in the FLOT group and 21 patients (52.5%) in the mDCF group (*p* = 0.001). TRG criteria also showed a higher rate of partial and complete response (TRG 1, 2, 3) in the FLOT group, with 44 patients (88%) achieving this compared to 21 patients (52.5%) in the mDCF group (*p* = 0.001). Complete radiological response according to RECIST criteria was achieved in 25 patients (50%) in the FLOT group and 11 patients (27.5%) in the mDCF group (*p* = 0.006). Table 1 shows the comparison of treatment response factors in the two groups.

## 4 Discussion

Although numerous guidelines have been recommended for the treatment of GC, many people worldwide continue to lose their lives to this disease. In 2020, approximately 1.1 million new cases and 770 000 deaths were reported due to GC disease. However, a new treatment regimen called the FLOT regimen has been used for GC patients, and the results have been promising [26].

Farrokhi et al. conducted a retrospective cohort study on 152 patients to investigate the most efficient neoadjuvant regimen among FOLFOX, DCF, ECF, and FLOT regimens. The results showed that the FLOT regimen had the highest efficacy for neoadjuvant chemotherapy compared to other regimens for GC in both OS and progression‐free survival (PFS) [27]. In the study by Wong et al., the performance of CF and mDCF regimens in advanced or locally recurrent GC patients was compared, and the mDCF regimen presented significant results for prolonging OS, PFS, and objective response rate (ORR) compared to CF [28]. Based on these recent studies, this study used FLOT and mDCF as two of the most effective regimens.

Our study evaluated the response to treatment using RECIST Ver 1.1, TRG, and R0 resection criteria. The study of Farrokhi et al. presented the highest rate of complete response (CR) in 3.1% of the FLOT group and 5.9% of the DCF group [27]. Zhang et al. reported that 13% of patients treated with the neoadjuvant FLOT regimen achieved CR [29]. In our study, the rate of radiologic CR was 50% in patients treated with the FLOT regimen.

In a clinical trial conducted by Al Batran et al., 265 patients were included to compare the histopathological regression following chemotherapy with FLOT and ECF/ECX regimens before surgery. 128 patients were treated with the FLOT regimen, and 137 were treated with the ECF/ECX regimen. The results showed that the FLOT regimen provided a pathologic complete response (TRG1) in 16% of patients [30]. A study conducted by Paszt et al. on the same subject as Al Batran's found that only six patients (13.95%) out of 53 in the FLOT group achieved TRG1 [31]. In our study, 13 patients (26%) achieved TRG1, which is similar to the results reported in Al Batran's study.

Our study included patients with a lower level of tissue invasion (T_IB_–T_IV_ compared to T_III_–T_IV_ in other studies), which may explain the better response to treatment, both radiologically and pathologically, compared to other studies.

In the study by Farrokhi et al., the FLOT neoadjuvant chemotherapy produced R0 resection in 93.8% and the DCF regimen in 94.1% of cases, the highest rate compared to other regimens [27]. In the study of Schulz et al., similar to ours, 50 patients who received neoadjuvant chemotherapy with the FLOT regimen were studied, and a rate of 86% for R0 resection was seen [32]. Our study showed a higher rate of R0 resection (80%) in the FLOT group, which is consistent with the findings of Schulz et al.

Furthermore, our study showed a significant reduction in disease stage, lymphatic involvement, and tissue infiltration in patients treated with the FLOT regimen compared to the mDCF regimen, which was also seen in similar studies on FLOT [33].

One significant difference between the FLOT and mDCF regimens is the use of oxaliplatin instead of cisplatin in the FLOT regimen. Huang et al. compared the safety and effect of cisplatin‐based and oxaliplatin‐based regimens; it has been seen that although the use of oxaliplatin reduces the possibility of stomatitis, anemia, neutropenia, alopecia, nausea, thromboembolism, and renal failure in comparison with cisplatin‐based therapy, this regimen led to an increased risk of thrombocytopenia and neuropathy. Also, the oxaliplatin‐based regimen has been associated with better OS, PFS, and ORR than cisplatin‐based regimens [34]. We observed that neuropathy was more evident in patients receiving the FLOT regimen, which is in line with the previous study by Huang et al. [34] and it might be due to the use of oxaliplatin in this regimen. However, unlike that study, we found that grade 3–4 thrombocytopenia was more common in the mDCF group.

Al Batran et al. identified neutropenia as the most common nonsurgical grade 3–4 adverse event of the FLOT regimen, affecting 67 patients (52%) out of 128 [30]. In the study by Farrokhi et al., the rate in the FLOT regimen was 37.2%, while the highest incidence of grade 3–4 neutropenia was observed in the DCF regimen at 51% of patients [27]. In our study, consistent with the Farrokhi et al. research, grade 3–4 neutropenia (ANC < 1000/μL) was more prevalent in the mDCF regimen, affecting 62.5% of patients. This may be attributed to the use of cisplatin instead of oxaliplatin in the mDCF regimen.

In the study by Al Batran et al., only 5% of patients experienced febrile neutropenia; however, in our study, this adverse effect was observed in 76% of patients [30]. Docetaxel, one of the drugs used in both regimens, is associated with myelosuppression and febrile neutropenia as side effects [30]. In our study, the dose of docetaxel was 60 mg/m^2^, which was higher than the dose used in Al Batran's study. Additionally, the 2‐week intervals between chemotherapies, shorter than the 3‐week intervals of the mDCF regimen, made the patients more susceptible to adverse effects.

Another difference between the two regimens is the use of leucovorin in the FLOT regimen. Leucovorin is a 5‐formyl derivative of folic acid, which, in combination with 5‐fluorouracil or methotrexate, is used in chemotherapy regimens. Studies suggest that its combination with 5‐fluorouracil increases the efficacy and toxicity, while some studies show less toxicity in this combination [35, 36]. The use of leucovorin in the FLOT regimen might be one reason for the higher response rate of this regimen compared to mDCF in our study.

One limitation of our study is that we did not examine OS in patients. Considering the results of the study by Castellanos et al. [37] and Li et al. [38], as well as the studies that focused on measuring OS in GC patients, it can be concluded that patients receiving the FLOT regimen have a higher chance of R0 resection for tumour removal. Therefore, the probability of overall survival is also higher for these patients [27]. In this study, the histological subtypes and differentiation status of GC have not been examined, which may lead to bias in the results. Another limitation of this study was that, due to the lack of permanent availability of medication and the potential for intermittent supply in our country, randomization methods could not be employed, resulting in an unavoidable selection bias under these circumstances. Compared to other studies on GC, our population was limited, which can be a drawback of our study. Future studies, by including larger populations, may further investigate this subject and compare the overall survival (OS) of patients and the toxicity of drugs across different histopathologies and differentiation statuses of GC. In our study, the effects of Body Mass Index (BMI), gender, and demographic variables have not been compared across the two groups, like many significant studies in the literature. However, according to several studies documented in the literature, these variables, although limited in scope, may influence the response rate of the participants [27, 39]. We recommend that future studies assess the effects of demographic variables on gastric cancer patients undergoing chemotherapy.

Our findings suggest that neoadjuvant chemotherapy using the FLOT regimen, when compared to the mDCF regimen, shows greater efficacy in cases of GC with local invasion that are eligible for surgery. The FLOT regimen has demonstrated fewer complications and greater effectiveness.

## Author Contributions

**Mehdi Pourghasemian:** conceptualization, project administration, supervision, validation, visualization. **Maryam Salimi:** data collection, writing draft. **Effat Iranijam:** supervision, visualization, data collection. **Mohammad Negaresh:** statistical analysis, writing – original draft, review, and editing.

## Ethics Statement

This study was approved by the Ethics Committee of Ardabil University of Medical Sciences with the Code of Ethics IR.ARUMS.REC 1399.572.

## Consent

Informed consent was obtained from the patients and/or guardian(s).

## Conflicts of Interest

The authors declare no conflicts of interest.

## Data Availability

The datasets used and/or analyzed during the current study are available from the corresponding author upon reasonable request.
